# Comparison Between 5- and 1-Year Outcomes Using Cutoff Values of Pressure Drop Coefficient and Fractional Flow Reserve for Diagnosing Coronary Artery Diseases

**DOI:** 10.3389/fphys.2021.689517

**Published:** 2021-07-14

**Authors:** Rupak K. Banerjee, Sruthi Ramadurai, Shreyash M. Manegaonkar, Marepalli B. Rao, Sathyaprabha Rakkimuthu, Mohamed A. Effat

**Affiliations:** ^1^Department of Mechanical and Materials Engineering, University of Cincinnati, Cincinnati, OH, United States; ^2^Research Services, Veteran Affairs Medical Services, Cincinnati, OH, United States; ^3^Department of Mechanical and Materials Engineering, University of Cincinnati, Cincinnati, OH, United States; ^4^Department of Environmental and Public Health Sciences, University of Cincinnati, Cincinnati, OH, United States; ^5^Department of Cardiology, University of Cincinnati Medical Center, Cincinnati, OH, United States

**Keywords:** pressure drop coefficient (CDP), fractional flow reserve (FFR), interventional cardiology, intermediate coronary stenosis, microvascular diseases, MACE, survival analysis

## Abstract

**Background:**

The current pressure-based coronary diagnostic index, fractional flow reserve (FFR), has a limited efficacy in the presence of microvascular disease (MVD). To overcome the limitations of FFR, the objective is to assess the recently introduced pressure drop coefficient (CDP), a fundamental fluid dynamics-based combined pressure–flow index.

**Methods:**

We hypothesize that CDP will result in improved clinical outcomes in comparison to FFR. To test the hypothesis, chi-square test was performed to compare the percent major adverse cardiac events (%MACE) at 5 years between (a) FFR < 0.75 and CDP > 27.9 and (b) FFR < 0.80 and CDP > 25.4 groups using a prospective cohort study. Furthermore, Kaplan–Meier survival curves were compared between the FFR and CDP groups. The results were considered statistically significant for *p* < 0.05. The outcomes of the CDP arm were presumptive as clinical decision was solely based on the FFR.

**Results:**

For the complete patient group, the %MACE in the CDP > 27.9 group (10 out of 35, 29%) was lower in comparison to the FFR < 0.75 group (11 out of 20, 55%), and the difference was near significant (*p* = 0.05). The survival analysis showed a significantly higher survival rate (*p* = 0.01) in the CDP > 27.9 group (*n* = 35) when compared to the FFR < 0.75 group (*n* = 20). The results remained similar for the FFR = 0.80 cutoff. The comparison of the 5-year MACE outcomes with the 1-year outcomes for the complete patient group showed similar trends, with a higher statistical significance for a longer follow-up period of 5 years.

**Conclusion:**

Based on the MACE and survival analysis outcomes, CDP could possibly be an alternate diagnostic index for decision-making in the cardiac catheterization laboratory.

**Clinical Trial Registration:**

www.ClinicalTrials.gov, identifier NCT01719016.

## Introduction

The impediment of blood flow to the heart muscle results from the sum of epicardial stenosis resistance and microvascular disease (MVD) resistance, which act in series (Pijls et al., 1993; Chilian, 1997; Kern, 2000; Fearon, 2004; Siebes et al., 2004). Delineation of the relative contributions of these resistances is vital, as they should be used to guide the clinical decision-making for the selection of the most appropriate treatments (Fearon, 2003; Verna et al., 2006). Diagnostic parameters such as fractional flow reserve (FFR, the ratio of the mean distal coronary and aortic pressure at hyperemia) and coronary flow reserve (CFR; the ratio of coronary flow at hyperemia to that at rest) are often used for the functional (hemodynamic) evaluation of stenosis in current clinical practice (Kern et al., 2006; Smith et al., 2006; Wijns and Kolh, 2010). Simultaneous FFR and CFR assessments are recommended to dissociate the severity of proximal epicardial coronary stenoses from distal MVD.

Currently, FFR (Pijls et al., 1995, 1996; Kern, 2000) is considered as gold standard and is commonly used in assessing the severity of intermediate coronary stenosis. A FFR less than 0.75 (Pijls et al., 1993) was shown to indicate a hemodynamic significance of coronary stenosis in a single vessel disease and 0.80 for multi-vessel disease (Pijls et al., 1995, 2007, 2010; Silber et al., 2005; Tonino et al., 2009, 2010; De Bruyne et al., 2014; Shlofmitz and Jeremias, 2017). Limited data exist for patients with other disease conditions, e.g., MVD. Furthermore, for intermediate stenosis that lie in the “gray” zone of 0.75–0.8 FFR value (Fearon and Yeung, 2003; Pijls, 2003), significant uncertainties exist. A decrease in flow across a stenosis resulting from concomitant MVD can elevate the distal coronary pressure and, with it, the true FFR value (Hoffman, 2000; Pijls et al., 2000; Meuwissen et al., 2002; Tamita et al., 2002; Chamuleau et al., 2003; De Bruyne, 2003; Park et al., 2016; Stegehuis et al., 2018). Thus, it is argued by these studies that, in the setting of MVD, a reduced flow and pressure drop across the stenosis results in an overestimated FFR. This, in turn, may erroneously indicate that the stenosis is insignificant and that PCI is not needed. Thus, caution is exercised by clinicians when applying available data to such patients. The FFR has yet to achieve a Class I recommendation in the current AHA/ACC guidelines (Fihn et al., 2014).

Using fundamental fluid dynamics principles and applying an analytical–numerical approach on published patient data, we developed (Banerjee et al., 2007) a combined pressure–flow functional parameter, pressure drop coefficient (CDP), which is the ratio of pressure drop across a stenosis to distal dynamic pressure (a measure of flow or velocity) to simultaneously detect stenosis and MVD. The CDP was tested *in vitro* (Sinha Roy et al., 2008; Peelukhana et al., 2009) as well as *in vivo* in animal studies (Banerjee et al., 2009; Kolli et al., 2011, 2012; Peelukhana et al., 2012, 2014b) and was able to differentiate between stenosis and MVD. Subsequently, we tested them in single- and two-center cohort patient groups (Kolli et al., 2014a,b, 2016; Peelukhana et al., 2014a, 2015, 2018; Effat et al., 2016; Hebbar et al., 2017) using prospective trials. In line with our studies, the importance of a combined approach of assessing both coronary pressure and flow has been reported by others as basis for the understanding of coronary physiology (van de Hoef et al., 2012; Shlofmitz and Jeremias, 2017; Gould, 2018; Stegehuis et al., 2018). In recent years, CDPs have been recognized as possible alternate coronary diagnostic parameters by other researchers (Govindaraju et al., 2014; Drenjancevic et al., 2015; Fearon, 2015; Johnson et al., 2015; Garcia et al., 2019).

First, the CDP was assessed to evaluate different degrees of stenosis severity in a patient population (Kolli et al., 2014b). An equivalent cutoff of CDP > 27.9 was established in relation to FFR < 0.75 (Kolli et al., 2014a, 2016) as a marker for single-vessel significant stenosis. Subsequently, our previous 1-year outcome study (Effat et al., 2016) showed that the percent major adverse cardiac events (%MACE) in the CDP > 27.9 group was lower when compared to the FFR < 0.75 group. The results of the survival analysis suggested that the survival time for the CDP > 27.9 group was significantly higher when compared to the FFR < 0.75 group. The results were similar for a FFR cutoff of 0.80 and associated CDP cutoff of 25.4. The MVD sub-group analysis (Hebbar et al., 2017) for %MACE and survival analysis showed similar trends in favor of CDP for both CFR < 2.0 and diabetic sub-groups. Both of these sub-groups were correlated with possible MVD in literature (Feigl, 1983; Muller et al., 1996; Miura et al., 2003; Bagi et al., 2004; Pijls et al., 2007; Picchi et al., 2010; Johnson et al., 2012). Additionally, CDP was correlated with hyperemic microvascular resistance (HMR) – another index that uses both pressure and flow measurements to evaluate MVD.

The objective of the current study is to compare (a) the 5-year outcome (%MACE and survival) between the CDP > 27.9 and FFR < 0.75 groups and (b) the outcomes between 1 year (Effat et al., 2016) and 5 years for the complete patient group. Extending our earlier 1-year (Hebbar et al., 2017) MVD sub-group analysis, this study also compares (a) the 5-year outcomes between the CDP > 27.9 and FFR < 0.75 for CFR < 2.0 and the diabetic patient sub-groups and extracted from the complete patient data (Effat et al., 2016) analyzed previously and (b) outcomes between 1- and 5-year patient sub-groups. All comparisons were also repeated for the FFR cutoff of 0.80 and CDP cutoff of 25.4.

## Materials and Methods

The protocol (Kolli et al., 2014b) for the study was approved by the institutional review board (IRB 2013-1256) at the University of Cincinnati Medical Center (UCMC) and Cincinnati Veterans Affairs Medical Center (CVAMC), and informed consent was obtained from all participants. Patients who underwent exercise testing and myocardial perfusion scans were consented based on the inclusion and exclusion criteria, as given below. A total of 86 patients who enrolled at UCMC and CVAMC constituted the study population. The clinical characteristics of the enrolled patients are summarized in Table 1.

**TABLE 1:** Summary of clinical data and characteristics of the 86 recruited patients.

**Variable**	**Study/group**
Sex (M/F)	77/9
Age (year)	61 ± 9
Ejection fraction (%)	58 ± 10
**Clinical history**	
Diabetes	42/86
Hypertension	70/86
Dyslipidemia	60/86
Previous myocardial infarction	21/86
Smoking history	52/86
Family history of CAD	23/86
LV hypertrophy	4/86
**Affected artery**	
LAD	43
LCX	17
RCA	26

*M, Male; F, Female; CAD, Coronary artery disease; LV, Left ventricle; LAD, Left anterior descending; LCX, Left circumflex; RCA, Right coronary artery.*

The receiver operating characteristic (ROC) curve was developed to determine the diagnostic performance of CDP, as reported in our previous studies (Kolli et al., 2014a, 2016). The ROC curves were generated, and the area under the curve (AUC) was calculated (MedCalc, version 10.2.0.0) using sensitivity and specificity values. The accuracy of a diagnostic test is obtained by AUC analysis. While an area of 0.5 represents a bad test, an area of 1 represents a perfect test. To correctly predict the outcome, the accuracy of the CDP was calculated for predefined and clinically used cutoff values of FFR (0.75 and 0.80) and CFR (2.0). The results were considered statistically significant for *p* < 0.05.

**FIGURE 1:**
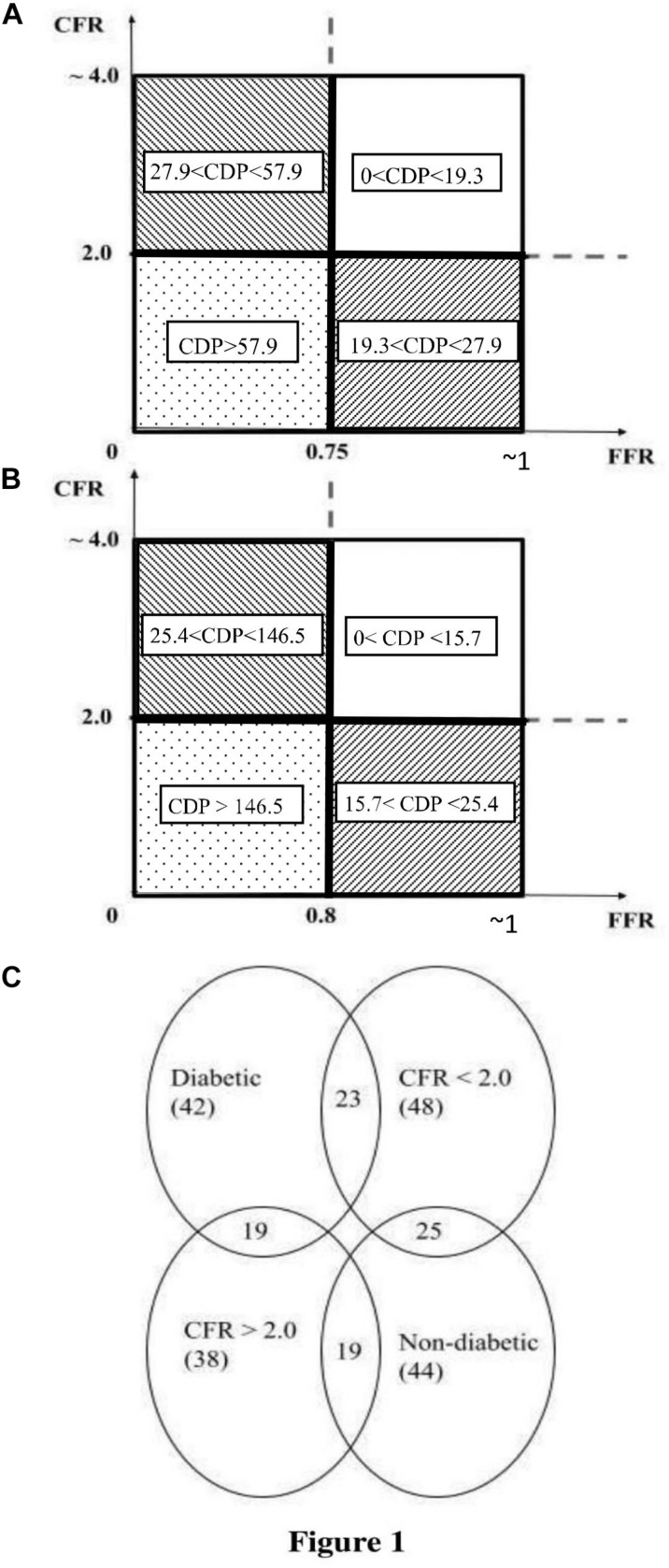
**(A)** Schematic representation of the pressure drop coefficient (CDP) cutoff for FFR = 0.75 and CFR = 2.0 (Kolli et al., 2014a). **(B)** Schematic representation of CDP cutoff for FFR = 0.80 and CFR = 2.0 (Kolli et al., 2016). **(C)** Venn diagram of the MVD sub-groups extracted from the total population of 86 patients. The figure shows the number of patients in each sub-group and highlights the intersection between the diabetic and CFR < 2.0 sub-groups. MVD, microvascular disease. CFR, coronary flow reserve; FFR, fractional flow reserve.

The consolidated cutoff values of CDP are shown in Figure 1A (CDP cutoffs based on FFR = 0.75 and CFR = 2.0) and Figure 1B (CDP cutoffs based on FFR = 0.8 and CFR = 2.0). The two different diseases (epicardial stenosis and MVD) lead to four possible combinations: (1) absence of both diseases, (2) absence of epicardial stenosis and presence of MVD, (3) presence of epicardial stenosis and absence of MVD, and (4) presence of both diseases based on established cutoff values of FFR (0.75 and 0.8) and CFR (2.0) (Kolli et al., 2014a, 2016). The FFR negative (−ve) group is indicative of (a) FFR > 0.75 when the FFR cutoff is 0.75 and (b) FFR > 0.8 when the FFR cutoff is 0.8 (Kolli et al., 2014a). Out of these 86 patients, 20 are FFR < 0.75 (+ve cases), and the remaining 66 are FFR > 0.75 (−ve cases). For an analogous scenario, 35 are CDP > 27.9 (+ve cases), and the remaining 51 are CDP < 27.9 (−ve cases). On a similar note, out of the same 86-patient group, 33 are FFR < 0.80 (+ve cases), and the remaining 53 are FFR > 0.80 (−ve cases). Correspondingly, 39 are CDP > 25.4 (+ve cases), and the remaining 47 are CDP < 25.4 (−ve cases).

The inclusion criteria for the study were (1) chest pain, (2) abnormal stress test, (3) an approximately 50% diameter stenosis (by visual assessment) in a major coronary artery on angiogram, and (4) left ventricular ejection fraction >25%. The exclusion criteria were (1) left ventricular ejection fraction <25%, (2) history of type II heparin-induced thrombocytopenia, (3) ostial lesions and serial stenoses, (4) significant left main stenosis, (5) non-dialysis-dependent chronic kidney disease with baseline serum creatinine greater than 2.5 g/dl, (6) significant co-morbid conditions that make coronary angiography prohibitive and contraindicated, and (7) pregnant women.

### Cardiac Catheterization and Pressure–Velocity Measurement

Using standard-of-care catheterization techniques, intra-coronary pressure–flow measurements across the stenosis were obtained by using either (1) a 0.014-in.-diameter guide wire (Combowire, Philips Corporation, CA, United States) that combines a standard Doppler sensor at the tip and a standard pressure sensor 1.5 cm proximal to the tip or (2) 0.014-in.−diameter pressure and Doppler guide wires separately. The sensor-tipped guide wire was (a) set at zero, (b) calibrated, (c) advanced through the guiding catheter, and (d) normalized to the aortic pressure at the ostium. Subsequently, it was introduced into the coronary artery and positioned distal to the stenosis in the target vessel, with the pressure transducer at least 3.0 cm distal to the stenosis. The position of the Doppler sensor was adjusted until a stable and optimal velocity signal was obtained distal to the stenosis (Kolli et al., 2016). The diameter of the Combowire and the pressure wire was the same, i.e., 0.014 in. The only difference between these sensor-tipped guide wires is that the Combowire has two (pressure and flow) sensors, whereas the pressure wire has a single (pressure) sensor. Both the Combowire and the pressure wire would have the same flow–obstruction effect since their diameters are identical. All signals were recorded continuously at rest and throughout induction as well as the decline of maximal hyperemia.

### CDP Formulation

Pressure drop coefficient (Banerjee et al., 2007) is defined as the ratio of *trans-*stenotic pressure drop to the distal dynamic pressure.

$$\mathrm{CDP}=\Delta \,P/\left(0.5\times \rho \times {\mathrm{APV}}^{2}\right)$$

where Δ*P* = *P*_a_ – *P*_d_ is the pressure drop across the stenosis, *P*_a_ and *P*_d_ are the mean pressures measured proximal and distal to the stenosis at hyperemia, respectively, and the distal dynamic pressure is the product of blood density (*ρ*) and the square of the average peak flow velocity (APV) distal to the stenosis at hyperemia at a constant value of 0.5. The *ρ* was assumed to be a constant (1.05 g/cm^3^), as it does not change significantly during the hyperemic (high shear) condition (Banerjee et al., 2009; Effat et al., 2016; Hebbar et al., 2017). Therefore, CDP represents the ratio between the total pressure drop (viscous loss plus loss due to momentum change with pressure recovery) and the dynamic pressure during blood flow.

### Data Access and Analysis

The 5-year follow-up for all 86 patients of this prospective cohort study was done through chart review and/or phone call. Out of 86 patients, 55 patients’ records were obtained from the computerized patient record system at CVAMC. The records of the other 31 patients were obtained from EPIC, the electronic health record system at UCMC. At 5 years, the primary outcomes, consisting of major adverse cardiac events (MACE), were determined. The 5-year outcomes are summarized in Table 2. The sub-group analysis for patients suffering from possible MVD was performed by extracting two sub-groups from the complete patient population: one sub-group consisted of patients with an abnormal CFR value (CFR < 2.0), and the other sub-group consisted of diabetic patients. Therefore, the MVD sub-groups are subsets extracted from the main set of 86 patients. Figure 1C shows the number of patients in the MVD sub-groups. It also highlights the overlap of the diabetic and CFR < 2.0 sub-groups. The 1-year outcomes of the same 86-patient cohort group (Effat et al., 2016) and sub-groups (Hebbar et al., 2017) were previously published.

**TABLE 2:** Summary of the %MACE outcomes for the recruited patients at 5-year follow-up.

**All patient**	**FFR < 0.75**	**CDP > 27.9**	**FFR > 0.75**	**CDP < 27.9**	**FFR < 0.80**	**CDP > 25.4**	**FFR > 0.80**	**CDP < 25.4**
All-cause mortality	5/20	6/35	9/66	8/51	8/33	7/39	6/53	7/47
Myocardial infarction	5/20	5/35	3/66	3/51	6/33	5/39	2/53	3/47
Revascularization	4/20	2/35	5/66	7/51	6/33	2/39	3/53	7/47
Total MACE	11/20	10/35	14/66	15/51	16/33	11/39	9/53	14/47
**CFR < 2.0**								
All-cause mortality	4/14	5/26	6/34	5/22	6/22	6/29	4/26	4/19
Myocardial infarction	3/14	3/26	1/34	1/22	3/22	3/29	1/26	1/19
Revascularization	2/14	2/26	3/34	3/22	3/22	2/29	2/26	3/19
Total MACE	7/14	8/26	9/34	8/22	10/22	9/29	6/26	7/19
**Diabetic**								
All-cause mortality	3/11	5/16	5/31	3/26	5/14	5/19	3/28	3/23
Myocardial infarction	5/11	5/16	2/31	2/26	5/14	5/19	2/28	2/23
Revascularization	3/11	2/16	4/31	5/26	4/14	2/19	3/28	5/23
Total MACE	8/11	9/16	9/31	8/26	11/14	9/19	6/28	8/23

*FFR < 0.75 vs. CDP > 27.9 and FFR < 0.80 vs. CDP > 25.4 are shown enclosed in thick borders.*

*MACE, Major adverse cardiac events; FFR, Fractional flow reserve; CDP, Pressure drop coefficient.*

### Statistical Analysis

For the outcome analysis for the complete patient group, the %MACE in the FFR < 0.75 group (*n* = 20) were compared against the %MACE in the CDP > 27.9 group (*n* = 35). Then, for each MVD sub-group, the %MACE in the FFR and CDP groups were compared. The same analysis for complete patient group and sub-group analysis was repeated for the FFR cutoff of 0.80 and equivalent CDP cutoff of 25.4. All statistical comparisons were performed using chi-square test. In addition, the 5-year %MACE outcomes were compared with the 1-year outcomes reported in our earlier study (Effat et al., 2016; Hebbar et al., 2017).

Furthermore, a survival analysis was done by generating Kaplan–Meier survival curves and comparing the long-term event-free survival of the FFR < 0.75 group and the CDP > 27.9 group. The analysis was done for the complete patient group and for both MVD sub-groups. The same procedure was repeated for the FFR cutoff of 0.80 and corresponding CDP cutoff of 25.4. The time duration between the index procedure and the time when the patient was last followed up was recorded. Any patient who reached the primary outcome (MACE) was counted as positive. Patients lost to follow-up or who did not reach the outcome were considered as censored data. The survival curves generated were compared for statistically significant difference using the log-rank test.

All analyses were performed using the statistical computing software R (Foundation for Statistical Computing, Vienna, Austria). The results were considered statistically significant for *p* < 0.05.

## Results

Comparisons of the 5-year outcomes between (a) the FFR and CDP groups and the (b) 1-year outcomes for complete patient group and MVD sub-groups for cutoff values FFR = 0.75 (i.e., CDP = 27.9) and FFR = 0.80 (i.e., CDP = 25.4) are discussed below.

### Percent MACE Outcomes for FFR < 0.75 and CDP > 27.9

#### Complete Patient Group

**FIGURE 2:**
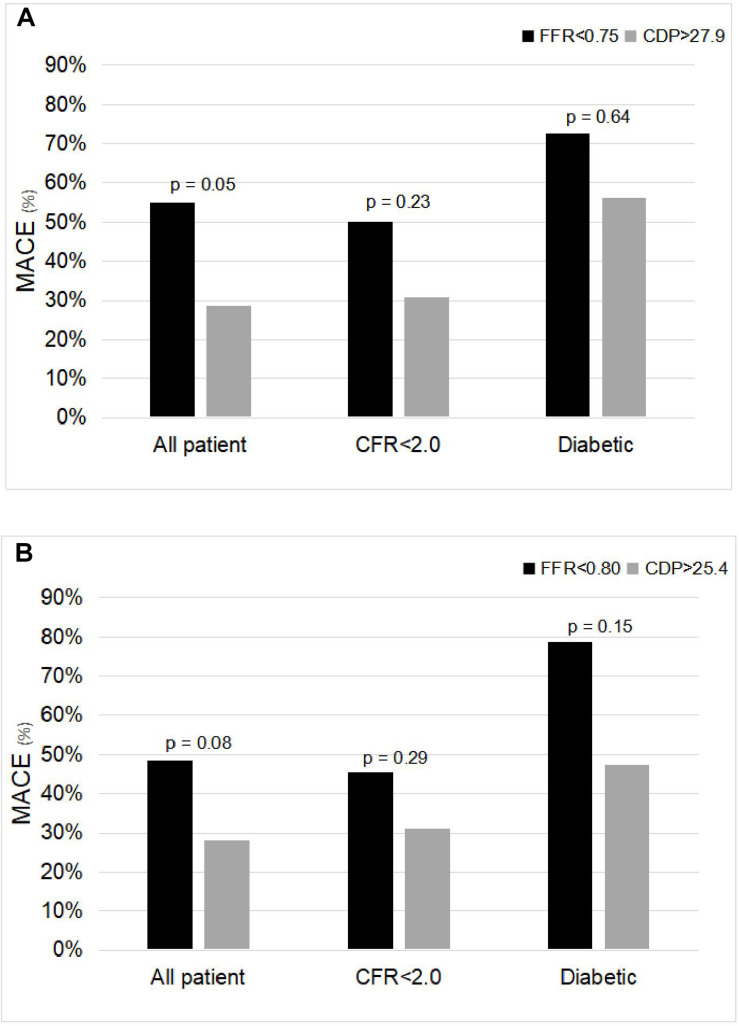
Comparison of %MACE between **(A)** FFR < 0.75 and CDP > 27.9 groups and **(B)** FFR < 0.80 and CDP > 25.4 groups for the all-patient group and for the microvascular disease sub-groups: CFR < 2.0 and diabetic. The *p*-values are provided for the chi-square test. MACE, major adverse cardiac events; FFR, fractional flow reserve; CDP, pressure drop coefficient.

The comparison of %MACE outcomes between the FFR < 0.75 and CDP > 27.9 (Banerjee et al., 2009) groups for the complete patient group and for the CFR < 2.0 and diabetic patient MVD sub-groups is shown in Figure 2A. For the complete patient group, the %MACE outcomes in the CDP > 27.9 group (10 out of 35, 29%) were lower than the corresponding values for the FFR < 0.75 group (11 out of 20, 55%), and the difference was near significant based on chi-square test (*p* = 0.05).

#### MVD Patient Sub-Groups

Similar trends were observed in the two MVD sub-groups. For the CFR < 2.0 sub-group, the %MACE outcomes in the CDP > 27.9 group (eight out of 26, 31%) were lower than the corresponding values for the FFR < 0.75 group (seven out of 14, 50%). The results were not statistically significant based on chi-square test (*p* = 0.23). For the diabetic sub-group, the %MACE outcomes in the CDP > 27.9 group (nine out of 16, 56%) were lower than the corresponding values for the FFR < 0.75 group (eight out of 11, 73%). The results were not statistically significant based on chi-square test (*p* = 0.64). Considering the trend, it is expected that statistical significance will improve with increased sample size.

### Percent MACE Outcomes for FFR < 0.80 and CDP > 25.4

#### Complete Patient Group

The analysis above was also repeated for the FFR cutoff of 0.80 and equivalent CDP cut-off of 25.4 (Kolli et al., 2011). Figure 2B shows the comparison of the %MACE outcomes between the FFR < 0.80 and CDP > 25.4 groups for the complete patient group and for the MVD sub-groups. For the complete patient group, the %MACE outcomes in the CDP > 25.4 group (11 out of 39, 28%) were lower than the corresponding values for the FFR < 0.80 group (16 out of 33, 48%), and the difference was borderline significant based on chi-square test (*p* = 0.08).

#### MVD Patient Sub-Groups

Similar trends were observed in the two MVD sub-groups. For the CFR < 2.0 sub-group, the %MACE outcomes in the CDP > 25.4 group (nine out of 29, 31%) were lower than the corresponding values for the FFR < 0.80 group (10 out of 22, 45%). The results were not statistically significant based on chi-square test (*p* = 0.29). For the diabetic sub-group, the %MACE outcomes in the CDP > 25.4 group (nine out of 19, 47%) were lower than the corresponding values for the FFR < 0.80 group (11 out of 14, 79%). The results were not statistically significant based on chi-square test (*p* = 0.15).

### Percent MACE Outcome Comparison Between 5 Years and 1 Year

**FIGURE 3:**
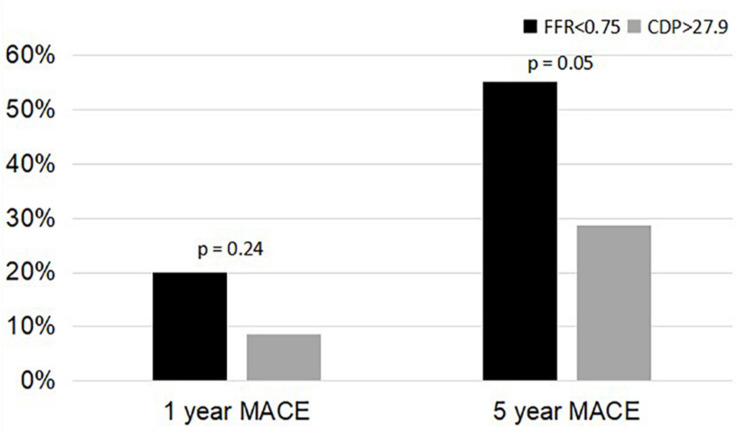
Comparison of %MACE between the FFR < 0.75 group and the CDP > 27.9 groups at 1 and 5 years. MACE, major adverse cardiac events; CDP, pressure drop coefficient; FFR, fractional flow reserve.

**FIGURE 4:**
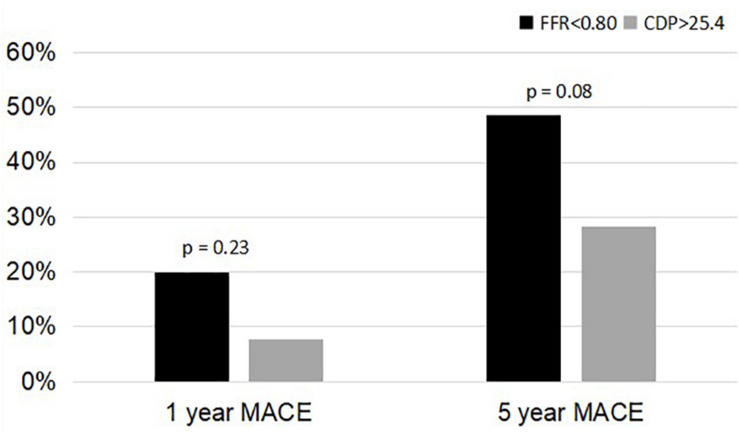
Comparison of %MACE between the FFR < 0.80 group and the CDP > 25.4 groups at 1 and 5 years. MACE, major adverse cardiac events; CDP, pressure drop coefficient; FFR, fractional flow reserve.

A comparison of the 5-year %MACE outcomes with the 1-year outcomes for the complete patient group published in our earlier study (Effat et al., 2016; Hebbar et al., 2017) shows that, for the longer follow-up period, the outcomes are consistent with the 1-year outcomes (Figures 3, 4). Not only are the trends consistent but also there is an improvement: for the FFR cutoff of 0.75, the *p*-values have reduced to the extent that the results are now near significant. Similarly, for the FFR cutoff of 0.80, the *p*-value is now borderline significant.

### Percent MACE Outcomes for FFR-Negative Groups

The %MACE outcomes between the FFR negative groups were also compared using chi-square test. Referring to Table 2, the %MACE in the FFR > 0.75 group (14 out of 66, 21%) and the CDP < 27.9 group (15 out of 51, 29%) were not significantly different (*p* = 0.31). Similarly, the %MACE in the FFR > 0.80 group (nine out of 53, 17%) and CDP < 25.4 group (14 out of 47, 30%) were not significantly different (*p* = 0.13).

The 5-year %MACE outcomes above suggest that if CDP-based interventional decisions were to be made, the %MACE outcomes would possibly be reduced when compared to the current FFR-based interventions.

### Survival Analysis for FFR < 0.75 and CDP > 27.9

#### Complete Patient Group

**FIGURE 5:**
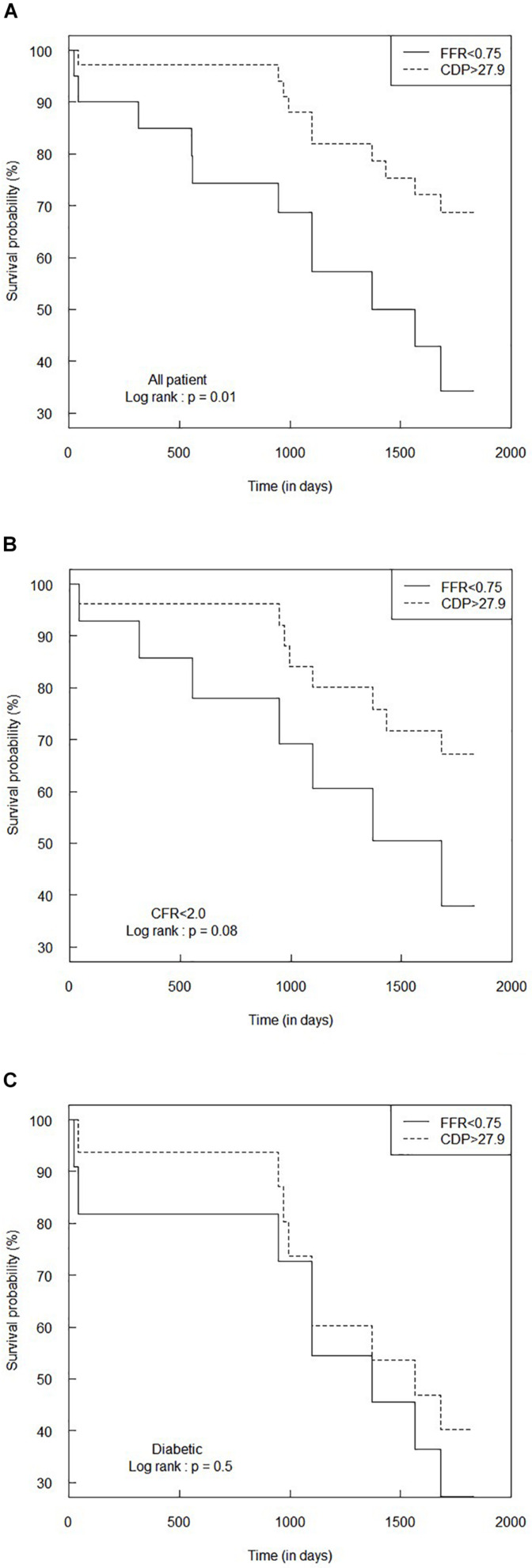
Comparison of Kaplan–Meier survival curves between FFR < 0.75 and CDP > 27.9 groups for **(A)** the all-patient group, **(B)** CFR < 2.0 sub-group, and **(C)** diabetic sub-group. The *p*-values are provided for the log-rank test. CDP, pressure drop coefficient; FFR, fractional flow reserve.

Figure 5A shows the Kaplan–Meier survival analysis performed for the complete patient group for the FFR cutoff of 0.75. The hazard ratio was calculated to be 0.35 (95% CI: 0.15–0.83). This means that the survival probability in the FFR < 0.75 group is 0.35 times the corresponding survival probability in the CDP > 27.9 group. Another way of interpretation is that the rate of adverse events in the CDP > 27.9 group is only 0.35 times the rate of hazard in the FFR < 0.75 group. The survival time for the CDP > 27.9 group (*n* = 35) was significantly higher (*p* = 0.01) compared to the FFR < 0.75 group (*n* = 20).

#### MVD Patient Sub-Groups

Figures 5B,C summarize the survival analysis for the abnormal CFR (<2.0) sub-group and the diabetic sub-group, respectively, for the FFR cutoff of 0.75. For the CFR < 2.0 sub-group, the hazard ratio was computed to be 0.42 (95% CI: 0.15–1.17), implying a trend toward higher survival expectancy in the CDP > 27.9 group. The survival time for the CDP > 27.9 group (*n* = 26) was higher than that of the FFR < 0.75 group (*n* = 14), and the difference was nearly significant (*p* = 0.08). For the diabetic sub-group, the hazard ratio was 0.72 (95% CI: 0.28–1.87). The survival time for the CDP > 27.9 group (*n* = 16) was not statistically different (*p* = 0.5) from the FFR < 0.75 group (*n* = 11).

### Survival Analysis for FFR < 0.80 and CDP > 25.4

#### Complete Patient Group

**FIGURE 6:**
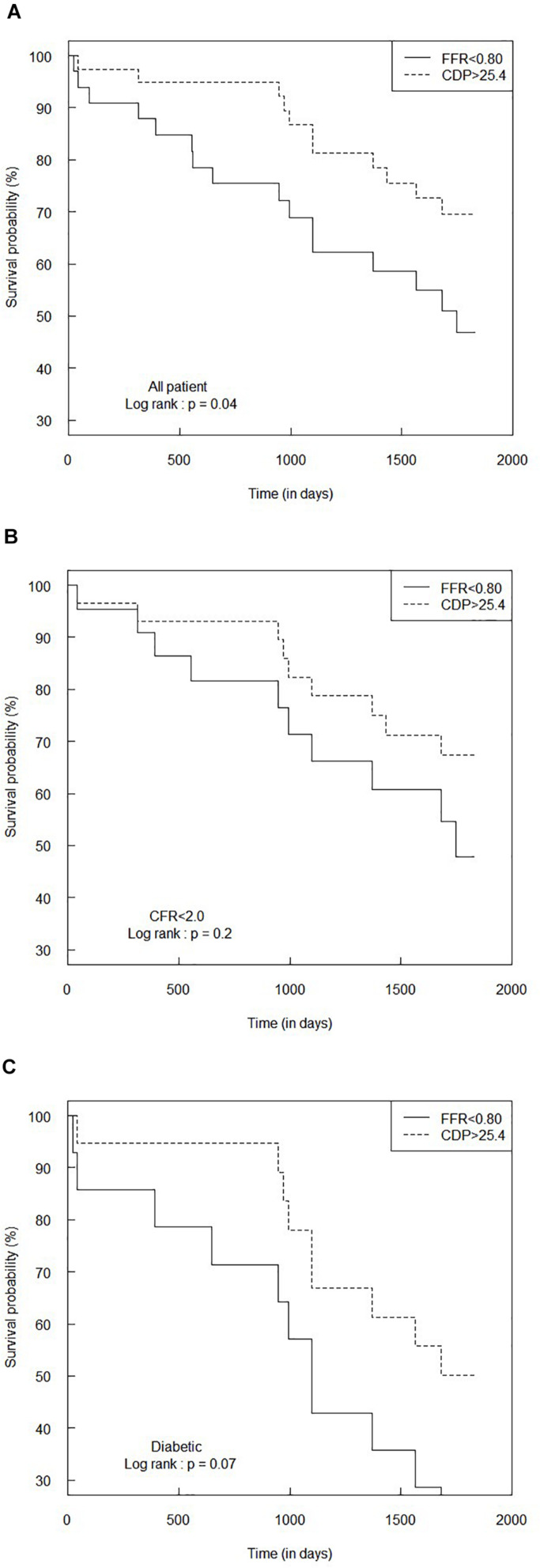
Comparison of Kaplan–Meier survival curves between FFR < 0.80 and CDP > 25.4 groups for **(A)** the all-patient group, **(B)** CFR < 2.0 sub-group, and **(C)** diabetic sub-group. The *p*-values are provided for the log-rank test. CDP, pressure drop coefficient; FFR, fractional flow reserve.

On a similar note, Figure 6A summarizes the Kaplan–Meier survival analysis for the complete patient group for the FFR cutoff of 0.80. The hazard ratio between the FFR < 0.80 and CDP > 25.4 groups was 0.46 (95% CI: 0.22–1.00), indicating that the rate of adverse events (hazard) in the CDP > 25.4 group is only 0.46 times the hazard rate in the FFR < 0.80 group. The survival time for the CDP > 25.4 group (*n* = 39) was significantly higher (*p* = 0.04) than that of the FFR < 0.80 group (*n* = 33).

#### MVD Patient Sub-Groups

Figures 6B,C summarize the results of the survival analysis for the CFR < 2.0 and diabetic sub-groups, respectively, for the FFR cutoff of 0.80. For the CFR < 2.0 sub-group, the hazard ratio was 0.56 (95% CI: 0.23–1.4). The survival time for the CDP > 25.4 group (*n* = 29) was not statistically different (*p* = 0.2) compared to the FFR < 0.80 group (*n* = 22). For the diabetic sub-group, the hazard ratio was 0.45 (95% CI: 0.19–1.1), indicating a marginally increased survival expectancy and a lower hazard rate in the CDP > 25.4 group. The difference in survival times between the CDP > 25.4 group (*n* = 19) and the FFR < 0.80 group (*n* = 14) was nearly significant (*p* = 0.07).

## Discussion

The advantages of combining both pressure and flow measurements within a single parameter is well supported by several published studies in the literature. In case of the current study, CDP, defined as coronary translesional pressure drop (Δ*p*) to distal dynamic pressure (0.5 × *ρ* × APV^2^), uses both pressure and flow measurements to assess stenosis severity in the presence or absence of MVD. The CDP is a non-dimensional parameter that originated from fundamental fluid dynamics principles. However, the use of such combined pressure–flow parameters under a clinical setting remained an unmet need.

The results of this 5-year outcome (MACE and event-free survival) analysis for the complete patient group suggest that, if a clinical decision is made on the basis of CDP relative to FFR, there would possibly be (a) reduced MACE and improved quality of life when comparing patients who had CDP > 27.9 to FFR < 0.75 and (b) a significant increase in event-free survival. The results for FFR using 0.80 as cutoff, with a corresponding CDP cutoff of 25.4, also resulted in similar outcomes. Interestingly, a comparison of the 5-year outcomes with the 1-year outcomes for the complete patient group showed similar trends with a marked improvement in the statistical significance for the 5-year follow-up period. The results of this 5-year outcome analysis for the MVD patient sub-groups (a) CFR < 2.0 and (b) diabetic patients show similar improved outcomes for CDP in relation to FFR and when compared with the 1-year outcomes.

In the presence of MVD, FFR, and CFR are affected in opposite directions. Consequently, assessment of ischemia by measuring FFR and CFR in the same stenosis, in the presence of MVD or diffused epicardial disease, may yield conflicting results in up to 40% of the cases (Johnson et al., 2012; Nishi and Fearon, 2019). This can be explained by the presence of MVD and/or diffuse epicardial disease that would reduce CFR, resulting in either increased FFR or insignificant influence on FFR. In contrast, a healthy microvascular auto-regulatory function may preserve CFR above the ischemic threshold, leading to abnormal FFR (Yokota et al., 2019). The complex interaction between pressure drop and flow in the presence of MVD or diffuse lesion might not be sufficiently explained by FFR or CFR alone. This is because FFR is a pressure-derived parameter, whereas CFR is a flow-derived endpoint. In contrast, CDP combines both the pressure drop and flow in a single parameter and therefore can delineate between concomitant stenosis and MVD (Kolli et al., 2012, 2014a,b, 2016).

It is well known that both FFR and CFR are significantly dependent on the attainment of peak hyperemia. Failure to achieve maximal hyperemia, as in the case of MVD, results in the inability to maintain a minimal constant microvascular resistance during the measurements (van de Hoef et al., 2014; Ahn et al., 2017; Garcia et al., 2019). This, in turn, results in the underestimation of pressure drop and overestimation of the FFR across a stenosis (Pijls et al., 2007). In the presence of MVD and a reduced level of hyperemia, the pressure drop and blood flow across stenosis are affected in the same direction. In case of such a concomitant disease, the contribution of reduced maximal hyperemic flow due to MVD is appreciably higher than that due to stenosis (Banerjee et al., 2009). Under such a scenario, the square of maximal hyperemic flow (APV^2^) in the denominator of CDP significantly accounts for the flow reduction, allowing an increased resolving power for CDP for the assessment of stenosis severity in the presence of MVD. Therefore, CDP can potentially have a significant advantage in clinical practice because it accounts for the dynamic pressure, a measure of velocity-square, in the denominator.

The use of dual sensor wires for coronary artery diagnostics has not gained sufficient traction in the cardiac catheterization laboratories. This is because of the added complexity in acquiring pressure–flow functional data. Nonetheless, as (a) the evidence from clinical outcome studies advances and (b) the technology progresses further in making the dual sensor wires more maneuverable, easier to use, and less expensive, the engagement of such novel and unique concepts will be more plausible for practice in the cardiac catheterization lab.

The clinical utility of FFR in applying a “functional” PCI approach for the treatment of stenosis, i.e., to only revascularize the angiographic stenosis that has significant FFR while deferring others, has been reported by several studies. For example, the DEFER study (Pijls et al., 2007) was comprised of 181 patients with stable ischemic heart disease and intermediate stenosis. The PCI was deferred for FFR > 0.75, and both arms were followed up with medical therapy. The rate of myocardial infarction (MI) or death was significantly lower in the deferred group when compared to the PCI group at 5-year follow-up. The FAME trial (Tonino et al., 2009, 2010; Pijls et al., 2010), comprising of 1,005 subjects, randomized the patients to either FFR- or angiography-guided PCI. The primary endpoint of MACE (MI, death, or repeat revascularization) at 1 year was significantly lower in the FFR-guided arm (13.2 vs. 18.3%, *p* = 0.02). The FAME-2 (De Bruyne et al., 2012; Xaplanteris et al., 2018) trial randomized 888 patients for comparing the outcomes between the FFR-guided PCI group and the optimal medical therapy group. Due to the significant difference in the primary endpoint of MACE in favor of the FFR-guided strategy, the study was terminated early. The results of these studies corroborate the importance of FFR in guiding clinical decision for the management of coronary artery disease.

In line with the above-mentioned studies, the current 5-year and previously published 1-year outcome studies purport an improved accuracy for CDP over FFR in predicting major ischemic events as well as angina-free survival. The reported outcome analysis corroborates the usefulness and advantage of CDP in decision-making with regard to deferment of revascularization during coronary artery procedures. Although statistical significance was not attained for some endpoints, the trends were uniformly consistent throughout both 5- and 1-year outcome follow-ups. More importantly, 5-year outcomes improved over 1-year follow-up. Further validation in a larger cohort and randomized groups with an extended follow-up period may yield an improved statistically significant difference in support of CDP.

### Limitations and Future Study

All the clinical decisions for this prospective cohort study were made on the basis of FFR only. The outcomes of the CDP arm were presumptive, as the clinical decision was solely based on the FFR. Furthermore, this study may be considered as an interim analysis because of a lower sample size. Therefore, (a) the use of a larger sample size and (b) a prospective randomized clinical trial of CDP vs. FFR are needed to further assess the clinical performance of CDP in relation to FFR and confirm the outcomes from this exploratory 5-year outcome study. Furthermore, there are several other hyperemic indices, e.g., index of myocardial resistance, hyperemic stenosis resistance, HMR, and resting indices, e.g., different diastolic resting indices to instantaneous wave-free ratio (iFR) that needs testing for delineation of MVD and stenosis (Van’t Veer et al., 2017; De Maria et al., 2020).

### Future Direction

The hypothesis need to be further tested using a two-sided χ^2^ test. Based on our prospective cohort study population consisting of 86 patients, the 1-year risk (conservative estimate) of MACE in our study for the FFR-guided and the CDP-guided groups was found to be 20.0 and 5.7%, respectively. Using a 0.05 level of significance, a power of 0.90, and a 14.3% difference [= 20–5.7% (Effat et al., 2016; Hebbar et al., 2017)] in MACE between the FFR group and the CDP group, at least 220 patients are required in the trial. Accounting for 10% mortality and missing data, ∼ 22 (= 10% of 220) more patients would be enrolled, leading to a total number of 242 (= 220 + 22) patients.

Extending the concept of CDP, a futuristic combined functional–anatomic parameter named lesion flow coefficient (Banerjee et al., 2007) (LFC), which accounts for mean pressure drop, mean coronary flow, and percentage area of stenosis, can be used in the future to assess the hemodynamic severity of a coronary artery stenosis in relation to MVD. The LFC, being a pressure–flow area parameter, is defined as the ratio of % area stenosis to the CDP_m_, which is the CDP measured at the stenosis throat:

$$\text{LFC}=\left[\left(1-k\right)/{\left({\mathrm{CDP}}_{\text{m}}\right)}^{0.5}\right]$$

where *k* is the lumen area ratio, (1−*k*) is percentage area of stenosis, and CDP_m_ is the pressure drop coefficient that includes contribution of pressure losses due to both momentum change and viscous dissipation at the stenosis throat during peak hyperemia. Importantly, the contribution of loss due to momentum change and viscous loss for variable lesion sizes can be distinguished using LFC. The concept of LFC stems from the fact that the CDP reaches an asymptotic value when the flow rate is very high, leading to a dominating loss due to momentum change, which is primarily the consequence of area change. Thus, at high flow rates, even a gradual (e.g., diffused) lesion behaves similar to a lesion with a sudden area change, e.g., focal stenosis. It is evident that the larger the severity of the diffused lesion and/or focal stenosis, e.g., for pre-angioplasty scenario, the higher the value of LFC. In other words, LFC decreases with lesser percentage area of stenosis, e.g., for post-angioplasty scenario, due to lower momentum change loss.

## Conclusion

The 5-year outcomes of this study indicate that patients in the CDP group had a reduced incidence of MACE and experienced an improved quality of life compared to the FFR group. CDP was also shown to be able to delineate epicardial stenosis in the presence of microvascular disease. Based on these outcomes and further validation in a larger randomized clinical trial, CDP could prove to be an effective and accurate diagnostic index for decision-making in the cardiac catheterization laboratory. Furthermore, combining CDP with FFR measurement could probably aid in managing patient outcome under a clinical setting.

## Data Availability Statement

The original contributions presented in the study are included in the article/supplementary material, further inquiries can be directed to the corresponding authors.

## Ethics Statement

The studies involving human participants were reviewed and approved by the Institutional Review Board (IRB 2013-1256) at the University of Cincinnati Medical Center (UCMC) and Cincinnati Veterans Affairs Medical Center (CVAMC). This study is registered with clinicaltrials.gov, the registration identification number is NCT01719016. The patients/participants provided their written informed consent to participate in this study.

## Author Contributions

SRD initially acquired the patient data for major adverse cardiac events and survival analysis by using chart review. Subsequently, ME (cardiologist, Director of the Cath Lab at University of Cincinnati Medical Center) and SRM independently verified the data. RB supervised the process of data acquisition. MR reviewed the statistical data analysis. RB and ME wrote the final version of the manuscript with the help of SM. All authors contributed to the article and approved the submitted version.

## Conflict of Interest

The authors declare that the research was conducted in the absence of any commercial or financial relationships that could be construed as a potential conflict of interest.

## Abbreviations

CDP: pressure drop coefficient
CFR: coronary flow reserve
CPRS: computerized patient record system
CVAMC: Cincinnati Veteran Affairs Medical Center
EPIC: electronic health record system
FFR: fractional flow reserve
HMR: hyperemic microvascular resistance
HSR: hyperemic stenosis resistance
iFR: instantaneous wave-free ratio
IMR: index of myocardial resistance
MACE: major adverse cardiac event
MVD: microvascular disease
PCI: percutaneous coronary intervention
UCMC: University of Cincinnati Medical Center.
